# Thirteenth Semi-Annual Meeting of the Central Texas Medical Association, Waco, Texas, January 9-10, 1900

**Published:** 1900-01

**Authors:** 


### Society Notes.

### Thirteenth Semi-Annual Meeting of the Central Texas Medical Association, Waco, Texas, January 9-10, 1900.

DEAR DOCTOR: The thirteenth semi-annual session of the Central Texas Medical Association will convene in the city of Waco, at 10 a. m., Tuesday and Wednesday, January 9th and 10th, 1900.

The accompanying program is a guarantee of a meeting of unusual interest. Attendance upon these meetings is a duty we owe to ourselves as well as to the profession. It elevates the standard of scientific medicine and promotes a general good feeling among all practitioners of the healing art.

Now, doctor, take a two days respite from your labors, come and meet your professional brethren, exchange experiences, get better acquainted and you cannot consider the time thus spent as lost. Our doors are always open to graduates in good standing. If you are not a member, let us insist that you visit the society and join during the present session.

Yours most respectfully and fraternally,

M. L. GRAVES, President.

N. A. OLIVE, Secretary.

PROGRAM.

TUESDAY, JANUARY 9, 1900. FORENOON SESSION, 10 A. M.

President's Address.

SECTION OF GENERAL MEDICINE.

H. C. Ghent, Chairman.
W. C. Blailock, Secretary.

1. "Typhoid Fever and Its Management." Paper by P. H. Brown, M. D., Troy, Ala. Discussion by Dr. J. C. J. King, Waco, and Dr. Daniel Parker, Calvert, Texas.

2. "Epilepsy." Paper by John S. Turner, M. D., First Assistant Superintendent Southwestern Insane Asylum, San Antonio, Texas. Discussion by Dr. O. I. Halbert, Waco, and Dr. J. D. Law, Salado, Texas.

3. "To Whom Does the Prescription Belong?" Paper by R. C. Fischer, Belton, Texas. Discussion by Dr. W. W. Greer, Cameron, and Dr. P. M. Kuykendall, Moody, Texas.

4. "Scarlet Fever: Differential Diagnosis." Paper by Garland B. Foscue, M. D., Waco, Texas. Discussion by Dr. H. C. Black, Waco, and Dr. W. E. Brown, Gatesville, Texas.

5. "Is Fever a Conservative Force?" Paper by J. W. Embree, M. D., Belton, Texas. Discussion by Dr. F. B. MacRae, Temple, and Dr. W. A. Wood, Hubbard City, Texas.

6. "Minor Surgery in Gynecology." Paper by Prof. W. H. Mathew, Kentucky School of Medicine, Louisville, Ky.

7. "The Relation of the Eye to the Kidney." Paper by Dr. John O. McReynolds, Dallas, Texas. Discussion by Dr. W. F. Cole, Waco, and Dr. Wm. Woodson, Temple, Texas.

8. "The Medical Treatment of the Acute Stage of Appendicitis." Paper by Dr. E. C. Garden, Lott, Texas. Discussion by Dr. W. R. Blailock, McGregor, and Dr. J. T. Valliant, Killeen, Texas.

9. "Diphtheria." Paper by Dr. W. M. Young, Dallas, Texas. Discussion by Dr. R. L. Kimmins and Dr. A. H. Snead, Waco, Texas.

10. "Something Needed for the Worthy Doctor to be Appreciated." Paper by R. H. Simpson, M. D., Turnersville. Discussion by Drs. W. E. Brown and J. H. Witt.

11. "The Ideal Doctor." Paper by N. A. Olive, M. D., Waco. Discussion by Drs. D. R. Wallace and A. M. Curtis.

12. "Variola." Paper by A. I. Cammack, M. D., Waco. Discussion by Drs. R. F. Minnock and O. I. Halbert.

13. "Continued or Typho Malarial Fever." Paper by W. A. Howard, M. D., Waco. Discussion by Drs. J. H. Sears and H. C. Black.

JANUARY 9, 1900. AFTERNOON SESSION, 2 P. M.

SECTION OF OBSTETRICS AND GYNECOLOGY.

Marvin B. Grace, M. D., Chairman.
A. R. Kuykendall, M. D., Secretary.

1. "Normal Labor and Its Treatment." Paper by O. I. Halbert, M. D., Waco. Discussion by Drs. M. T. McNeill and W. B. Blailock.

2. "The Retroposed Uterus; Its Cause and Treatment." Paper by C. E. Smith, M. D., Waco. Discussion by Drs. C. T. Young and Wallace Wilcox.

3. Conservative Gynecology." Paper by J. T. Harrington, M. D., Waco. Discussion by Drs. J. L. McGlasson and A. T. Ezell.

4. "An Asexual Monstrosity With Imperforate Anus and Other Unusual Deformities." Specimen presented by Dr. O. I. Halbert, Waco.

5. Electricity in Gynecology." Paper by M. B. Grace, M. D., Walnut. Discussion by Drs. A. M. Curtis and A. H. Snead.

6. "Instrumental Delivery." Paper by A. R. Kuykendall, M. D., Morgan. Discussion by Drs. J. D. Moore and J. E. Earl.

7. "Puerperal Septicæmia." Paper by J. H. Alexander, M. D., Meridian. Discussion by Drs. S. Gage and W. R. Thompson.

8. "Abortion, Spontaneous and Induced." Paper by Dr. J. A. Winfrey, Winnewood, Indian Territory.

JANUARY 9, 1900. EVENING SESSION, 8 P. M.

1. "Irresistable Impulse as a Defense in Criminal Prosecution." Paper by J. Walter Cocke, Esq., Waco. Discussion by Felix D. Robertson, Esq., and Dr. R. W. Park.

NOTE.—A Surgical Clinic will be held by Dr. J. W. Hale, at his office, from 12:30 to 2:30 daily.

WEDNESDAY, JANUARY 10, 1900. FORENOON SESSION 10 A. M.

Election of Officers.

SECTION OF SURGERY.

J. M. Frazier, M. D., Chairman.
J. S. McCelvey, M. D., Secretary.

1. "The Ideal Surgeon." Paper by J. M. Frazier, M. D., Belton. Discussion by Drs. A. C. Scott and A. M. Curtis.

2. "Hare Lip and Cleft Palate." Paper by E. C. Gordon, M. D., Lott. Discussion by Drs. Daniel Parker and Samuel E. Milliken.

3. "Appendicitis." Paper by J. W. Hale, M. D., Waco. Discussion by Drs. W. M. Young and A. H. Caldwell.

4. "Splenectomy for Floating Spleen—Report of a Case." Paper by Dr. A. C. Scott, M. D., Temple. Discussion by Drs. J. W. Miller and R. L. Kimmins.

5. "Gall Stones—Report of a Case." Paper by J. W. Hale, M. D. Discussion by Drs. A. E. Spohn and W. O. Wilkes.

Other important papers were received too late to get in the program.

SECOND DAY.—AFERNOON SESSION, 2 P. M.

SECTION OF OPHTHALMOLOGY AND OTOLOGY.

C. Guy Reily, M. D., Chairman.
J. R. Ferrel, M. D., Secretary.

1. "The Proper Mode of Testing the Eye and Adjusting Spectacles." Paper by C. Guy Reily, M. D., Waco. Discussion by Drs. J. R. Ferrel and J. M. Woodson.

2. "The Relation of the Eye to Intra-Cranial Affections." Paper by Jno. O. McReynolds, M. D., Dallas. Discussion by Drs. E. D. Capps and W. F. Cole.

3. "Operations in the Nose." Paper by W. F. Cole, M. D., Waco. Discussion by Drs. R. P. Talley and M. M. Smith.

4. "Cataract." Paper by J. R. Ferrel, M. D., Waco. Discussion by Drs. J. M. Woodson and John O. McReynolds.

A free Eye Clinic will be held daily from 9 a. m. to 10 a. m. by Drs. C. Guy Reily and J. R. Ferrel.

SECTION OF NERVOUS DISEASES.

M. L. Graves, M. D., Chairman.
F. R. Ross, Secretary.

"Criminal Responsibility of Epileptics." Paper by C. M. Rosser, M. D., Dallas.

"HEREDITY."

John Preston, M. D., Lockhart.

